# Supplementary material for: Functional Characterization of MdTAC1a Gene Related to Branch Angle in Apple (Malus x domestica Borkh.)
Source: Int J Mol Sci. 2022 Feb 7;23(3):1870. doi: 10.3390/ijms23031870 (PMC8836888; doi:10.3390/ijms23031870)
Supplement: Supplementary file 1 [file ijms-23-01870-s001.zip › ijms-1568608-supplementary.pdf]

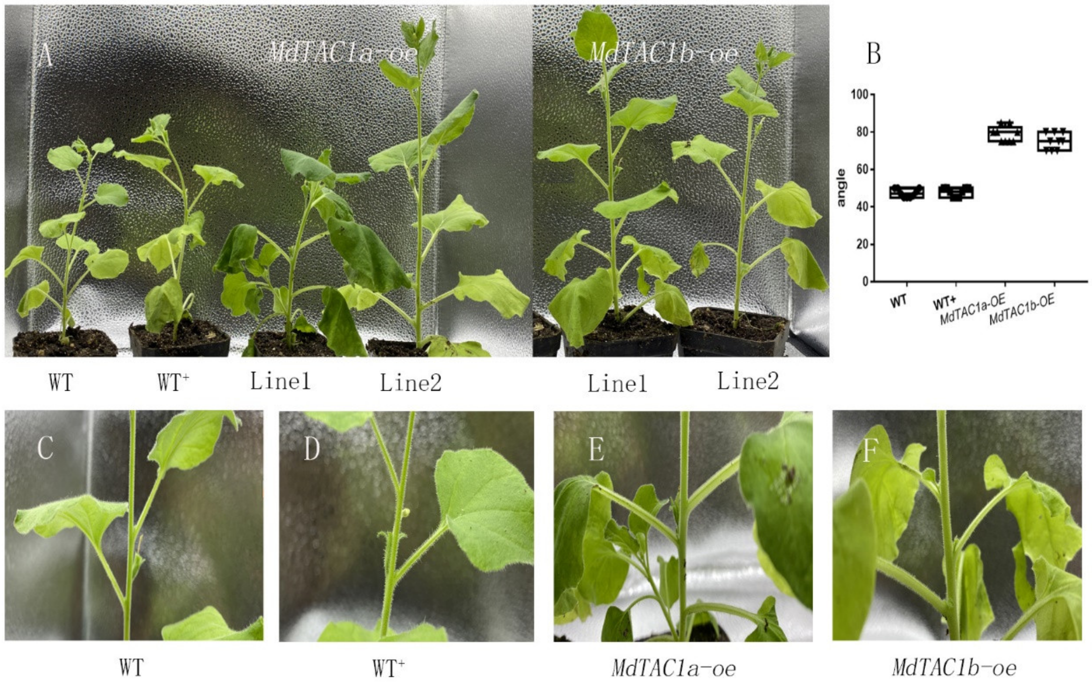

Figure S1 Phenotype analysis of *MdTAC1a* and *MdTAC1b* over-expressed in transgenic tobaccos at flowering (after 30d growth).

A, WT, negative, *MdTAC1a*-OE and *MdTAC1b*-OE plant growth state at the time after 30 days growth of flowering, C, D, E, F are the angle magnifications of the corresponding plants, and B is the angle statistics of the plants during this period.

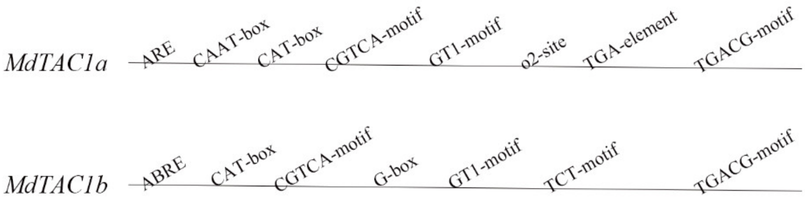

Figure S2. Cis-acting elements in CDS of *MdTAC1a* and *MdTAC1b*.

A

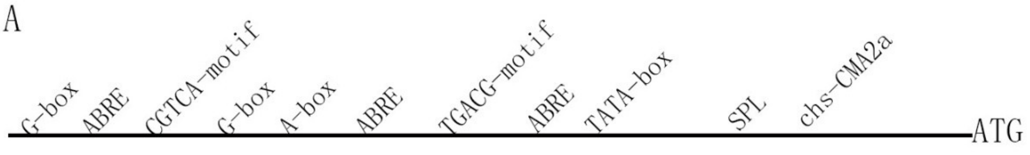

B

MASGHEVQVKLTSAHDLKNVNWRNGAVRPYAVVWVDPKRKCSSRVDEEGDTSPY

WDETLTIPLPGPVDGDTTLHVDIVHAGSDPDTKPLIGSARLKLREVLDDVGFDE

RASRTLHLKRPSGRPHGKVDVKVTIRKPRPRVADPYYAPPYGVPPPAGSRDLPT

PPPAYGAPYGAPAPPAPYNPYYPAAPPSGYPYNGYNAAPPQYGQPAPYGQTGQG

SYGQPGQGYGQGSYGQEEKKKSKFGGMGTGTGLAVGAVAGVLGGLALAEGFEYM

EDKIADDAAEKVEDDLYDGDDY

Figure S3. cis-acting elements in promoters and domain in CDS of *MdSRC2*
A, the cis-acting element of *MdSRC2* gene in 2000bp upstream of ATG. B, the domain of the *MdSRC2* gene in the CDS region, where the underlined part is the domain C2 SUPERFAMILY.

Table S1. Statistics on branch angles of 11 cultivars of four tree types.

Tree Ideo Type	Cultivars	Branch Angles
Columnar-type	Wijcik	36.8±2.18
	Waltz	38.5±1.50
	Maypole	41.6±2.50
Standard-type	Bolero	40.2±1.84
	McIntosh	54.0±3.74
	Summerland McIntosh	55.8±4.56
	Fuji	58.0±6.40
Spur-type	Fukushima Spur	68.5±3.91
	Miyazaki Spur	67.3±2.28
	Mutsu Spur	69.0±5.83
Weeping-type	Granny Smith	86.0±4.36

Table S2. Primers used for Quantitative real-time PCR analysis.

Primer Name	Forward Primer	Reverse Primer
qPCR-*MdACTIN*	GGATTTGCTGGTGATGATGCT	AGTTGCTCACTATGCCGTGC
qPCR -*MdTAC1a*	ACTTGTTGATGTTGACGGGCTTGA	CCTGATCGTCCTCTTCGCTCTCC
qPCR -*MdTAC1b*	CACAAGGGCACACAAGCATTTCT	TCGTTCTGAGGGTTGAAGGGTTT
qPCR-*NbACTIN*	AGAGGTTCCGTTGCCCAGAAGT	GCTAGGAGCCAAAGCCGTGATT
qPCR-*NbGA20ox*	CATAGGCGATACATTTATGGCG	CAGCTCTGTAATGCTTTTGTGT
qPCR-*NbGA2ox*	GTGCTGGAAATGATAGCAGAAG	AAAAGGAGTAGTGATCAGGTGG
qPCR-*NbPT*	AACGCAAAGGTATCCCTCA	CATCAACCCAAATAAAGCAAC
qPCR-*NbPIN1*	ATTAGGAACCCGAACACTTA	ACACCCATTGAGAAAGCAG
qPCR-*NbPIN2*	TATTCTGTTCAATCGTCCCG	CCCGCTCATACTCCTACCTC
qPCR-*NbCCD7*	TTGGCTACGATTGGCAAAGTG	TTGGCGAGAACCAGAAGAAGT
qPCR-*NbARF2*	CACAAACAGGGTACAGCACTCG	AAATCTTGCGAACCATACCACA
qPCR-*NbARF3*	TTATCGTCGGTATTCAGGTTC	TCTCCCTTCCTACTGATGTCC
qPCR-*NbPHOT1*	AAGCCTCATAGGAAGGACAGC	GCATAGAGTGCGGGAAGAAAA
qPCR-*NbPHOT2*	CAATTAGCAGAAGCGGAGAAA	CAGTCATCGTAAAGAAGCCAC
qPCR-*NbIAA5*	GCCTTTGAGAATGGCAGTGAA	CGCAAGCCTTAAACGGTATCT

Table S3. Primers used for *MdTAC1a/b*–GFP fusion constructs in this study.

Primer Name	Sequence (5′ to 3′)
MdTAC1a-F	ATGAAGATCTTCAACTGGGTTCATAAGC
MdTAC1a-R	TCAATGGACACAAGCAGTAGCACCTTG
MdTAC1b-F	ATGAAGATCTTCAACTGGGTTCATAAGC
MdTAC1b-R	TCAATGGACACAAGCAGTAGCACCTTG
BamHI-*MdTAC1a*	ggactcggtacccggggatccATGAAGATCTTCAACTGGGTTCATA
SaLl-*MdTAC1a*-R	acccccctccgccaccgtcgacATGGACACAAGCAGTAGCACCTT
BamHI-*MdTAC1b*-F	ggactcggtacccggggatccATGAAGATCTTCAACTGGGTTCATA
SaLl-*MdTAC1b*-R	acccccctccgccaccgtcgacATGGACACAAGCAGTAGCACCTT

Table S4. Primers used for promoter amplification.

Primer Name	Sequence (5′ to 3′)
MdTAC1a-pro-F	TTGCCAGTAACCCAACACCAATCAT
MdTAC1a-pro-R	ATGTGCATGGTTCTGCAACTAGTAC
MdTAC1b-pro-F	ATGGTATACGAACAGTCGCCAGAAG
MdTAC1b-pro-R	TCTGATGAAGCCGCTTATGAACC
InDel-*MdTAC1a*-F	GGTTAAATTAAGGTTAGCCCAATTC
InDel-*MdTAC1a*-R	AATTATCAGAGCAAAGCCCCAG

Table S5. Primers used for Y_2H, BiFC analysis, and Co-IP in this study.

Primer Name	Sequence (5′ to 3′)
T7	TAATACGACTCACTATAGGGCG
3AD	GAGATGGTGCACGATGCACAG
EcoRI-*MdTAC1*-BD-F	atggccatggaggccgaattcATGAAGATCTTCAACTGGGTTCATA
EcoRI-*MdTAC1*-BD-R	ccgctgcaggtcgacggatccTCAATGGACACAAGCAGTAGCA
MdSRC2-F	ATGGCGTCCGGTCACGAA
MdSRC2-R	CTAGAAGTCATCGCCGTCGTCG
XmaI-*MdSRC2*-AD-F	ggccagtgaattccacccgggATGGCGTCCGGTCACGAA
SacI-*MdSRC2*-AD-R	attcatctgcagctcgagctcCTAGAAGTCATCGCCGTCGTCG
BiFC -C-xbaI-*MdTAC1a*-F	gagaacacgggggactctaga ATGAAGATCTTCAACTGGGTTCATAAGC
BiFC-C-xhoI-*MdTAC1a*-R	gacagtactatcgatggatcc TCAATGGACACAAGCAGTAGCACCTTG
BiFC-N-xbaI-*MdSRC2*-F	gagaacacgggggactctaga ATGGCGTCCGGTCACGAA
BiFC-N-xhoI-*MdSRC2*-R	gacagtactatcgatggatcc CTAGAAGTCATCGCCGTCGTCG
CoIP- *MdTAC1a*-HA-F	gagaacacgggggactctagaATGAAGATCTTCAACTGGGTTCATAA
CoIP- *MdTAC1a*-HA-R	gcccccggggtcgacggtaccATGGACACAAGCAGTAGCACCTT
CoIP- *MdSRC2*-FLAG-F	atacaccaaatcgactctagaATGGCGTCCGGTCACGAA
CoIP- *MdSRC2*-FLAG-R	catggtaccggatccactagtGTAGTCATCGCCGTCGTACAGA
